# Supplementary material for: Drug repurposing for aging research using model organisms
Source: Aging Cell. 2017 Jun 16;16(5):1006–15. doi: 10.1111/acel.12626 (PMC5595691; doi:10.1111/acel.12626)
Supplement: Supplementary file 7 — Data S1 Zip‐Archive of all report cards. [file ACEL-16-1006-s007.zip › RC_AGS.pdf]

AGS

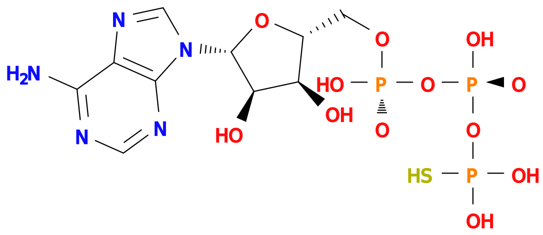

Database identifiers

ChEMBLCompound	CHEMBL131890
CHEBI	27575
eMolecules	29936603

Ranking

	Rank	Score
Drosophila	276/697	0.6
C. elegans	519/591	0.056

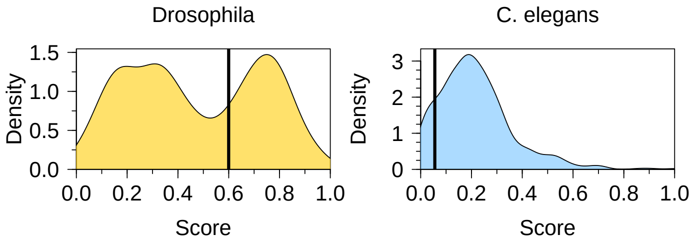

	Ageing implication	Domain conservation	Binding site conservation	Binding affinity	Bioavailability	Lipinski	Promiscuity	Purchasability	Drug approval	**Total**
Drosophila	1.0	0.975	1.0	0.801	(0.9)	-0.15	-0.054	0.1	0.0	0.6
C. elegans	1.0	0.974	1.0	0.801	0.204	-0.15	-0.054	0.1	0.0	0.056

Names

- **adenosine 5'-[gamma-thio]triphosphate**
- (gamma-S)ATP
- Adenosine 5'-O-(3-thiotriphosphate)
- Atpgammas
- gamma-Thio-ATP
- Phosphothiophosphoric acid-adenylate ester

Roles

ChEBI entry 27575 has no roles

Status

Approved drug (according to ChEMBL)	No
Number of Rule of 5 violations	3
Binding affinity to original target in log units (RF-Score prediction)	6.39
Burns *C. elegans* bioavailability prediction	-7.71

Compound Target Characteristics

Heat shock protein HSP 90-alpha

Best gene implication in ageing for this target family came from gene Q18688 annotated in UniProt release 2014_02. Annotation GO 8340 (determination of adult lifespan) was Inferred from Genetic Interaction

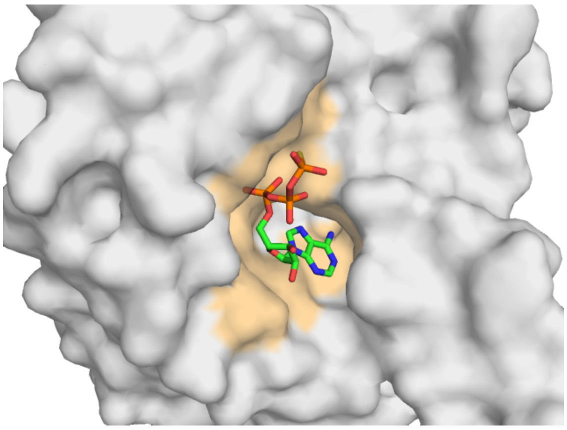

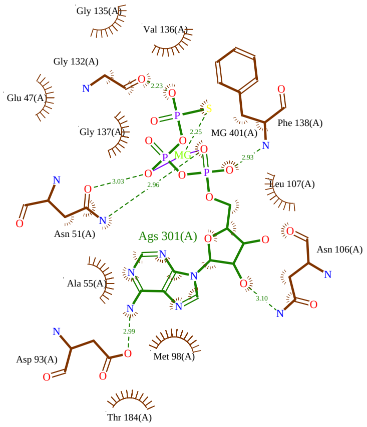

protein	amino acids contacts (binding site)
PDB:3t2s:chainA:P07900	E N A D M N L G G V G F T
tr:K9JA46:K9JA46_HUMAN	E N A D M N L G G V G F T
sp:P07900:HS90A_HUMAN	E N A D M N L G G V G F T
tr:Q6B437:Q6B437_RAT	E N A D M N L G G V G F T
sp:P82995:HS90A_RAT	E N A D M N L G G V G F T
tr:B7ZC50:B7ZC50_MOUSE	E N A D M N L G G V G F T
tr:Q8C2A9:Q8C2A9_MOUSE	E N A D M N L G G V G F T
tr:Q3TKB9:Q3TKB9_MOUSE	E N A D M N L G G V G F T
tr:Q3TKG0:Q3TKG0_MOUSE	E N A D M N L G G V G F T
sp:P07901:HS90A_MOUSE	E N A D M N L G G V G F T
tr:Q80Y52:Q80Y52_MOUSE	E N A D M N L G G V G F T
sp:P02828:HSP83_DROME	E N A D M N L G G V G F T
sp:Q18688:HSP90_CAEEL	E N A D M N L G G V G F T
sp:P02829:HSP82_YEAST	E N A D M N L G G V G F T

	whole protein		domain-based		contact-based	
protein	ident	simil	ident	simil	ident	simil
PDB:3t2s:chainA:P07900	1.0	1.0	1.0	1.0	1.0	1.0
tr:K9JA46:K9JA46_HUMAN	1.0	1.0	1.0	1.0	1.0	1.0
sp:P07900:HS90A_HUMAN	1.0	1.0	1.0	1.0	1.0	1.0
tr:Q6B437:Q6B437_RAT	0.22	0.22	0.85	0.85	1.0	1.0
sp:P82995:HS90A_RAT	0.99	1.0	1.0	1.0	1.0	1.0
tr:B7ZC50:B7ZC50_MOUSE	0.27	0.27	0.86	0.86	1.0	1.0
tr:Q8C2A9:Q8C2A9_MOUSE	0.37	0.37	0.99	1.0	1.0	1.0
tr:Q3TKB9:Q3TKB9_MOUSE	0.75	0.76	0.99	1.0	1.0	1.0
tr:Q3TKG0:Q3TKG0_MOUSE	0.75	0.76	0.99	1.0	1.0	1.0
sp:P07901:HS90A_MOUSE	0.99	1.0	0.99	1.0	1.0	1.0
tr:Q80Y52:Q80Y52_MOUSE	0.99	1.0	0.99	1.0	1.0	1.0
sp:P02828:HSP83_DROME	0.77	0.92	0.85	0.96	1.0	1.0
sp:Q18688:HSP90_CAEEL	0.74	0.89	0.76	0.94	1.0	1.0
sp:P02829:HSP82_YEAST	0.6	0.85	0.68	0.9	1.0	1.0

Hsp83 (FBgn0001233) associated phenotypes

cell cycle defective, cell polarity defective, developmental rate defective, female semi-fertile, lethal - all die before end of pupal stage, lethal - all die during third instar larval stage, partially lethal, partially lethal - majority die, short lived

(Information from FlyBase)

Hsp83 (UniProt:P02828) annotation

Function: Molecular chaperone that promotes the maturation, structural maintenance and proper regulation of specific target proteins involved for instance in cell cycle control and signal transduction. Undergoes a functional cycle that is linked to its ATPase activity. This cycle probably induces conformational changes in the client proteins, thereby causing their activation. Interacts dynamically with various co-chaperones that modulate its substrate recognition, ATPase cycle and chaperone function. Together with Hop and piwi, mediates canalization, also known as developmental robustness, likely via epigenetic silencing of existing genetic variants and suppression of transposon-induced new genetic variation. Required for piRNA biogenesis by facilitating loading of piRNAs into PIWI proteins. (PubMed:21186352, PubMed:22902557).

Subunit: Homodimer (By similarity). Forms a complex with Hop and piwi; probably Hop mediates the interaction between piwi and Hsp83. Interacts with shu. (, PubMed:21186352, PubMed:22902557).

Subcellular location: Cytoplasm.

Induction: In contrast to other major heat shock proteins, this one is also expressed at normal growth temperatures. It is also developmentally expressed during oogenesis.

Domain: The TPR repeat-binding motif mediates interaction with TPR repeat-containing proteins.

(Information from UniProt)

daf-21 (WBGene00000915) associated phenotypes

chemotaxis variant, dauer constitutive, dauer recovery variant, larval lethal, lethal, odorant chemosensory response variant, sluggish, sterile

(Information from WormBase)

daf-21 (UniProt:Q18688) annotation

Function: Molecular chaperone that promotes the maturation, structural maintenance and proper regulation of specific target proteins involved for instance in cell cycle control and signal transduction. Undergoes a functional cycle that is linked to its ATPase activity. This cycle probably induces conformational changes in the client proteins, thereby causing their activation. Interacts dynamically with various co-chaperones that modulate its substrate recognition, ATPase cycle and chaperone function. By stabilizing the receptor-type guanylate cyclase daf-11 or another signal transduction component that regulates cGMP levels, plays a role in dauer formation and chemotaxis to non-volatile and volatile attractants detected by AWC sensory neurons (PubMed:10790386, PubMed:7828815). Participates in the control of cell cycle progression at the prophase/metaphase transition in oocyte development by ensuring the activity of wee-1.3 kinase, which negatively regulates cdk-1 through its phosphorylation (PubMed:16466390). Regulates yap-1 nuclear export after heat shock treatment (PubMed:23396260). (PubMed:10790386, PubMed:16466390, PubMed:23396260, PubMed:7828815).

Subunit: Homodimer. (UniProtKB:P08238).

Subcellular location: Cytoplasm, perinuclear region (PubMed:12950278). Note=Perinuclear region of somatic cells.

Tissue specificity: In the embryo comma stage, expression is strongly detected in cells of the head region and less so in other areas. In early larvae, expressed in postembryonic germ cells derived from Z2 and Z3 cells and the head region, in both hermaphrodites and males. Under heat stress conditions, larval expression is not only detected in germ cells, but also all over the body. In adult hermaphrodites, expression is localized uniquely in the germ cells. (PubMed:12950278).

Developmental stage: Highly expressed throughout development. (PubMed:10790386).

Domain: The TPR repeat-binding motif mediates interaction with TPR repeat-containing proteins.

(Information from UniProt)

Cyclin-dependent protein kinase PHO85

Best gene implication in ageing for this target family came from gene P48609 annotated in UniProt release 2014_02. Annotation GO 8340 (determination of adult lifespan) was Inferred from Mutant Phenotype

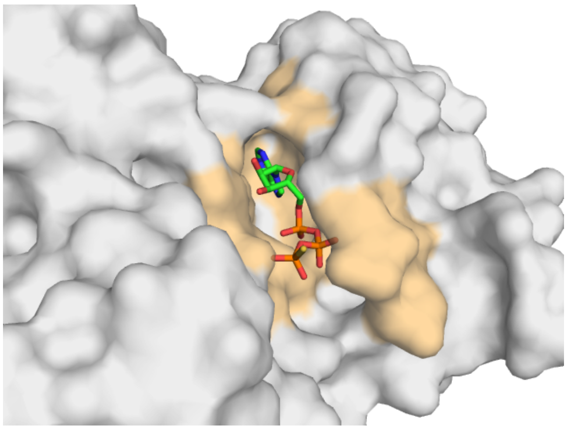

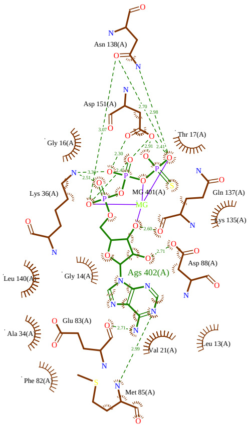

protein	amino acids contacts (binding site)																	
PDB:2pmi:chainA:P17157	-	-	-	-	-	A	V	A	K	F	E	M	D	D	K	Q	N	L	D
PDB:4krc:chainA:P17157	L	G	G	T	Y	A	V	A	K	F	E	M	D	D	K	Q	N	L	D
sp:Q00535:CDK5_HUMAN	I	G	G	T	Y	G	V	A	K	F	E	C	D	D	K	Q	N	L	D
sp:Q03114:CDK5_RAT	I	G	G	T	Y	G	V	A	K	F	E	C	D	D	K	Q	N	L	D
sp:P49615:CDK5_MOUSE	I	G	G	T	Y	G	V	A	K	F	E	C	D	D	K	Q	N	L	D
tr:Q543F6:Q543F6_MOUSE	I	G	G	T	Y	G	V	A	K	F	E	C	D	D	K	Q	N	L	D
sp:P48609:CDK5_DROME	I	G	G	T	Y	G	V	A	K	F	E	C	D	D	K	Q	N	L	D
tr:G5ECH7:G5ECH7_CAEEL	I	G	G	T	Y	G	V	A	K	F	E	C	D	D	K	Q	N	L	D
sp:P17157:PHO85_YEAST	L	G	G	T	Y	A	V	A	K	F	E	M	D	D	K	Q	N	L	D

	whole protein		domain-based		contact-based	
protein	ident	simil	ident	simil	ident	simil
PDB:2pmi:chainA:P17157	1.0	1.0	1.0	1.0	0.74	0.44
PDB:4krc:chainA:P17157	1.0	1.0	1.0	1.0	1.0	1.0
sp:Q00535:CDK5_HUMAN	0.54	0.82	0.55	0.83	0.84	0.87
sp:Q03114:CDK5_RAT	0.53	0.81	0.55	0.83	0.84	0.87
sp:P49615:CDK5_MOUSE	0.54	0.81	0.55	0.83	0.84	0.87
tr:Q543F6:Q543F6_MOUSE	0.54	0.81	0.55	0.83	0.84	0.87
sp:P48609:CDK5_DROME	0.51	0.8	0.52	0.81	0.84	0.87
tr:G5ECH7:G5ECH7_CAEEL	0.51	0.8	0.52	0.82	0.84	0.87
sp:P17157:PHO85_YEAST	1.0	1.0	1.0	1.0	1.0	1.0

Cdk5 (FBgn0013762) associated phenotypes

neuroanatomy defective, short lived

(Information from FlyBase)

Cdk5 (UniProt:P48609) annotation

Function: Probably involved in the control of the cell cycle. Interacts with D1 and D3-type G1 cyclins. Possible regulator of neuronal differentiation and/or development (By similarity).
Tissue specificity: Abundantly expressed in all adult tissues. Lower levels found in larvae and early embryos. Barely detectable in late embryos.

(Information from UniProt)

cdk-5 (WBGene00000407) associated phenotypes

aldicarb resistant

(Information from WormBase)

cdk-5 (UniProt:G5ECH7) annotation

Function: Proline-directed serine/threonine-protein kinase which, in several motor neurons, promotes the polarized trafficking of synaptic vesicles and dense-core vesicles (DCV). In the ventral nerve cord, phosphorylates lin-10 and thereby prevents lin-10- mediated anterograde trafficking of the glutamate receptor glr-1 (PubMed:17671168, PubMed:21609829). Involved in the inhibition of glr-1 trafficking in hypoxic conditions (PubMed:22252129). In DA motor neurons but not in DB motor neurons, regulates axonal transport of synaptic vesicle precursors by inhibiting dynein-mediated retrograde transport (PubMed:20510931). Regulates the trafficking of dense-core vesicles in DA and DB motor neurons by promoting anterograde trafficking to the axon and preventing dynein-dependent trafficking to the dendrite (PubMed:22699897). May regulate these processes in association with cdka-1/p35 (PubMed:17671168, PubMed:20510931). Activity may be regulated by cyy-1 (PubMed:20510931). Involved in synapse formation during DD motor neuron remodeling by regulating transport of disassembled synaptic material to the new synaptic sites probably by activating the motor protein unc-104/kinesin-3 (PubMed:21609829). Regulates microtubule polarity in the dendrite of DB motor neurons (PubMed:22699897). (PubMed:17671168, PubMed:20510931, PubMed:21609829, PubMed:22252129, PubMed:22699897).

Cofactor: Mg(2+) Evidence=(PubMed:20510931); Note=Binds 2 Mg(2+) ions. (UniProtKB:P24941);

Subunit: Heterodimer composed of a catalytic subunit cdk-5 and a regulatory subunit cdka-1. Interaction with cdka-1 is required for cdk-5 activation. (PubMed:22699897).

Subcellular location: Cytoplasm (PubMed:20510931). Cell projection, dendrite (PubMed:20510931). Note=Localizes predominantly to presynaptic sites and in dendrites as faint puncta. (PubMed:20510931).

Disruption phenotype: Several glr-1-dependent behaviors are affected including an absence of backward locomotion after nose- touching stimuli and a reduction in reverse locomotion (PubMed:17671168). In L4 mutants, incomplete elimination of ventral rab-3-positive synaptic vesicles associated with a delay in the formation of dorsal rab-3-positive synaptic vesicles in DD motor neurons. Normal formation of ventral synapses in DD motor neurons at the L1 stage (PubMed:21609829). In addition, mutants have an increase in anterograde dense-core vesicle trafficking and in the number of plus-end-out microtubules in DB motor neuron dendrites (PubMed:22699897). Reduced sensitivity to the acetylcholine esterase inhibitor aldicarb (PubMed:22699897). (PubMed:17671168, PubMed:21609829, PubMed:22699897).

(Information from UniProt)

Serine/threonine-protein kinase mTOR

Best gene implication in ageing for this target family came from gene Q9VK45 annotated in UniProt release 2014_02. Annotation GO 8340 (determination of adult lifespan) was Inferred from Mutant Phenotype

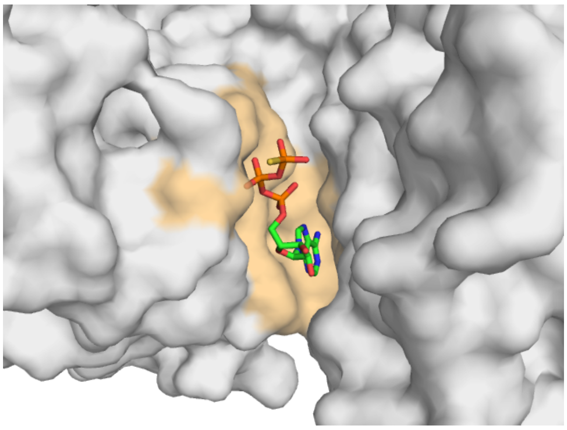

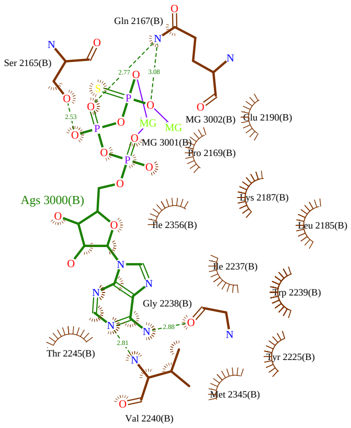

protein	amino acids contacts (binding site)
PDB:4jsp:chainB:P42345	S Q P L K E Y I G W V T M I
tr:B1AKP8:B1AKP8_HUMAN	S Q P L K E Y I G W V T M I
sp:P42345:MTOR_HUMAN	S Q P L K E Y I G W V T M I
sp:P42346:MTOR_RAT	S Q P L K E Y I G W V T M I
sp:Q9JLN9:MTOR_MOUSE	S Q P L K E Y I G W V T M I
tr:Q8T9I6:Q8T9I6_DROME	S Q P L K E Y I G W V T M I
tr:Q960H0:Q960H0_DROME	S Q P L K E Y I G W V T M I
tr:H1ZYC1:H1ZYC1_DROME	S Q P L K E Y I G W V T M I
sp:Q9VK45:TOR_DROME	S Q P L K E Y I G W V T M I
tr:H1ZYB7:H1ZYB7_DROME	S Q P L K E Y I G W V T M I
tr:H1ZYB5:H1ZYB5_DROME	S Q P L K E Y I G W V T M I
sp:Q95Q95:TOR_CAEEL	S Q P L K E Y I G W V T M I
sp:P32600:TOR2_YEAST	S Q P V K E Y L G W V T M I

	whole protein		domain-based		contact-based	
protein	ident	simil	ident	simil	ident	simil
PDB:4jsp:chainB:P42345	1.0	1.0	1.0	1.0	1.0	1.0
tr:B1AKP8:B1AKP8_HUMAN	0.3	0.3	1.0	1.0	1.0	1.0
sp:P42345:MTOR_HUMAN	1.0	1.0	1.0	1.0	1.0	1.0
sp:P42346:MTOR_RAT	0.99	1.0	0.99	1.0	1.0	1.0
sp:Q9JLN9:MTOR_MOUSE	0.99	1.0	1.0	1.0	1.0	1.0
tr:Q8T9I6:Q8T9I6_DROME	0.13	0.16	0.78	0.93	1.0	1.0
tr:Q960H0:Q960H0_DROME	0.2	0.26	0.78	0.93	1.0	1.0
tr:H1ZYC1:H1ZYC1_DROME	0.52	0.81	0.78	0.93	1.0	1.0
sp:Q9VK45:TOR_DROME	0.52	0.81	0.78	0.93	1.0	1.0
tr:H1ZYB7:H1ZYB7_DROME	0.52	0.81	0.78	0.93	1.0	1.0
tr:H1ZYB5:H1ZYB5_DROME	0.52	0.81	0.78	0.93	1.0	1.0
sp:Q95Q95:TOR_CAEEL	0.31	0.66	0.65	0.88	1.0	1.0
sp:P32600:TOR2_YEAST	0.39	0.71	0.65	0.89	0.86	0.98

Tor (FBgn0021796) associated phenotypes

body size defective, cell cycle defective, cell size defective, chemical sensitive, decreased cell number, decreased cell size, developmental rate defective, dominant, germline clone, gravitaxis defective, increased cell death, increased cell size, lethal - all die before end of pupal stage, lethal - all die before end of second instar larval stage, long lived, mitotic cell cycle defective, neuroanatomy defective, nutrition conditional, size defective, small body, somatic clone, some die during pupal stage, some die during second instar larval stage, some die during third instar larval stage, sterile

(Information from FlyBase)

Tor (UniProt:Q9VK45) annotation

Function: Promotes cell and tissue growth, maintains tissue homeostatis and controls responses to environmental stress and aging. Regulates growth during animal development by coupling growth factor signaling to nutrient availability. Central regulators of autophagy. May be involved in atg1 phosphorylation. May also be involved, directly or indirectly, in the control of neuronal function. Phosphorylates S6K/p70S6K, in vitro. May regulate the activity of S6K. Overexpression inhibits growth and reduces cell size. Affects the timing of neuronal cell differentiation. Hyperactivation of the signaling leads to accelerated differentiation, whereas inhibition of the signaling retards differentiation. Thus, in addition to controlling growth of the cell in which it resides, it can also influence growth of distant cells and organs during development via a humoral mechanism. In short, during development, it primarily controls growth, whereas in the adult, where there is relatively little growth, it controls aging and other aspects of nutrient-related physiology. Rag GTPases act as activators of TORC1 in response to amino acid signals. (PubMed:11069885, PubMed:11069888, PubMed:12559758, PubMed:14505573, PubMed:15454083, PubMed:16469695, PubMed:18604198, PubMed:19211682, PubMed:19225150).

Subunit: May be part of a minimal complex, TORC1, consisting oftor, raptor and lst8. May be part of a minimal complex, TORC2, consisting of tor, rictor and lst8 (By similarity). Self-

associates; assembles into homomultimeric complexes. Component of a multiprotein complex. (, PubMed:16219781).
Induction: By PI3K/Akt signaling, or by nutrients such as amino acids, and by high cellular energy levels. (PubMed:19211682).
Disruption phenotype: Not lethal. Displays phenotypes characteristic of amino acid deprivation, including reduced nucleolar size, lipid vesicle aggregation in the larval fat body, and a cell type-specific pattern of cell cycle arrest that can be bypassed by overexpression of the S-phase regulator cyclin E. Reach only the size of second instar larvae, at which point they undergo cell cycle arrest. (PubMed:11069885, PubMed:11069888).

(Information from UniProt)

let-363 (WBGene00002583) associated phenotypes
adult growth variant, late larval lethal, lethal

(Information from WormBase)

let-363 (UniProt:Q95Q95) annotation
Function: Kinase that regulates the mRNA translation machinery, probably by modulating the activity of translation factors such as eIF-4G and eIF-2. It may have some protein kinase activity instead of lipid kinase activity. May act as a mediator of lifespan regulation by insulin signaling and nutrient sensing. (PubMed:12225660, PubMed:14668850).
Subcellular location: Nucleus (PubMed:12225660).
Tissue specificity: Ubiquitous. Expressed in all major tissues and organs, including the intestine, gonads and hypodermal cells. (PubMed:12225660).
Disruption phenotype: Defects strongly increase lifespan, the mean lifetime being of 25 days instead of 10 days, suggesting that it may be involved in aging process. (PubMed:14668850).

(Information from UniProt)

Transitional endoplasmic reticulum ATPase

Best gene implication in ageing for this target family came from gene P54811 via mapping the annotation from Ensembl WBGene00007352 via mapping the annotation from EntrezGene 174624 via mapping the annotation from GenAgeModels 1759 annotated in GenAge release 17.

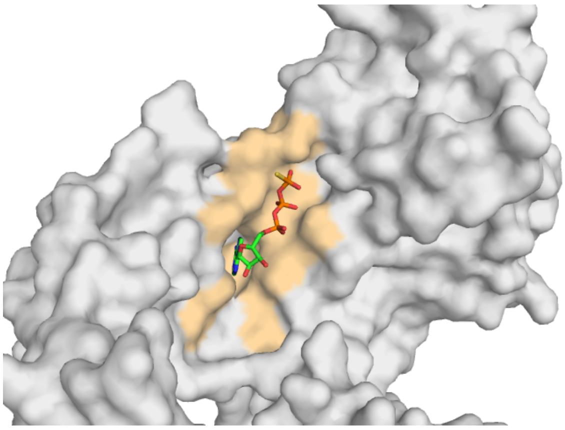

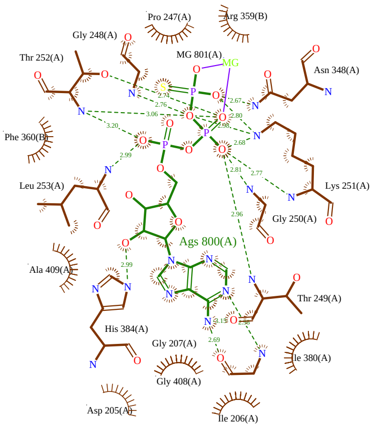

protein	amino acids contacts (binding site)
PDB:3hu1:chainA:P55072	D I G C P G T G K T L N R F I H G A
PDB:3hu2:chainA:P55072	D I G C P G T G K T L N R F I H G A
PDB:3hu3:chainA:P55072	D I G C P G T G K T L N R F I H G A
PDB:4kln:chainA:P55072	D I G C P G T G K T L N R F I H G A
PDB:4ko8:chainA:P55072	D I G C P G T G K T L N R F I H G A
tr:Q96IF9:Q96IF9_HUMAN	D I G C P G T G K T L N R F I H G A
sp:P55072:TERA_HUMAN	D I G C P G T G K T L N R F I H G A
sp:P46462:TERA_RAT	D I G C P G T G K T L N R F I H G A
tr:Q8BNF8:Q8BNF8_MOUSE	D I G C P G T G K T L N R F I H G A
sp:Q01853:TERA_MOUSE	D I G C P G T G K T L N R F I H G A
sp:Q7KN62:TERA_DROME	D I G C P G T G K T L N R F V H G A
tr:D0IQG4:D0IQG4_DROME	D I G C P G T G K T L N R F V H G A
sp:P54811:TERA1_CAEEL	D L G V P G T G K T L N R F I H G A
sp:P54812:TERA2_CAEEL	D L G V P G T G K T L N R F I H G A
sp:P25694:CDC48_YEAST	D I G C P G T G K T L N R F V H G A

	whole protein		domain-based		contact-based	
protein	ident	simil	ident	simil	ident	simil
PDB:3hu1:chainA:P55072	1.0	1.0	1.0	1.0	1.0	1.0
PDB:3hu2:chainA:P55072	1.0	1.0	1.0	1.0	1.0	1.0
PDB:3hu3:chainA:P55072	1.0	1.0	1.0	1.0	1.0	1.0
PDB:4kln:chainA:P55072	1.0	1.0	1.0	1.0	1.0	1.0
PDB:4ko8:chainA:P55072	1.0	1.0	1.0	1.0	1.0	1.0
tr:Q96IF9:Q96IF9_HUMAN	0.8	0.8	1.0	1.0	1.0	1.0
sp:P55072:TERA_HUMAN	1.0	1.0	1.0	1.0	1.0	1.0
sp:P46462:TERA_RAT	1.0	1.0	1.0	1.0	1.0	1.0
tr:Q8BNF8:Q8BNF8_MOUSE	0.9	0.9	1.0	1.0	1.0	1.0
sp:Q01853:TERA_MOUSE	1.0	1.0	1.0	1.0	1.0	1.0
sp:Q7KN62:TERA_DROME	0.83	0.94	0.88	0.97	0.94	0.98
tr:D0IQG4:D0IQG4_DROME	0.8	0.92	0.88	0.97	0.94	0.98
sp:P54811:TERA1_CAEEL	0.76	0.9	0.83	0.95	0.89	0.9
sp:P54812:TERA2_CAEEL	0.76	0.9	0.86	0.96	0.89	0.9
sp:P25694:CDC48_YEAST	0.66	0.87	0.83	0.96	0.94	0.98

TER94 (FBgn0261014) associated phenotypes

RU486 conditional, decreased cell death, flightless, increased cell death, learning defective, neuroanatomy defective, partially lethal - majority die, somatic clone, some die during pupal stage, stress response defective, temperature conditional

(Information from FlyBase)

TER94 (UniProt:Q7KN62) annotation

Function: Necessary for the fragmentation of Golgi stacks during mitosis and for their reassembly after mitosis. Involved in the formation of the transitional endoplasmic reticulum (tER). The transfer of membranes from the endoplasmic reticulum to the Golgi apparatus occurs via 50-70 nm transition vesicles which derive from part-rough, part-smooth transitional elements of the endoplasmic reticulum (tER). Vesicle budding from the tER is an ATP-dependent process. Involved in the ubiquitin-proteasome system. Important for oskar mRNA localization and/or anchoring during oogenesis. Involved in germ cell formation. (PubMed:14657277, PubMed:18590813).
Subunit: Homohexamer. Interacts with tud, vas, papi and AGO3.(PubMed:10564274, PubMed:18590813, PubMed:21447556).
Subcellular location: Cytoplasm. Nucleus. Note=Component of the meiotic nuage, also named P granule, a germ-cell-specific organelle required to repress transposon activity during meiosis.
Tissue specificity: Present in the mushroom bodies of the protocerebrum and in the glomeruli of the antennal lobe. Present in nurse cells, oocytes and sperm bundles (at protein level). (PubMed:10564274, PubMed:10656772, PubMed:9639875).
Developmental stage: Present in egg, pupa and imago but not larva (at protein level). (PubMed:9639875).

(Information from UniProt)

cdc-48.1 (WBGene00007352) associated phenotypes
tunicamycin hypersensitive, unfolded protein response variant

(Information from WormBase)

cdc-48.2 (WBGene00008053) associated phenotypes
reduced brood size

(Information from WormBase)

Dual specificity mitogen-activated protein kinase kinase 1

Best gene implication in ageing for this target family came from gene Q02750 annotated in UniProt release 2014_02. Annotation GO subterm of 7568 (aging) was Inferred from Mutant Phenotype

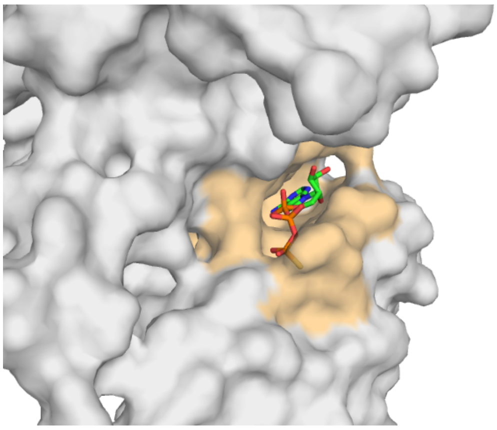

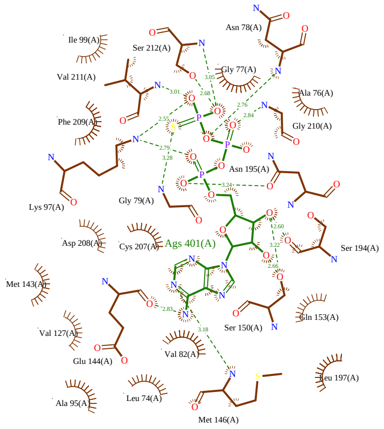

protein	amino acids contacts (binding site)																										
PDB:3eqc:chainA:Q02750	L	G	A	G	N	G	G	V	A	K	I	V	M	E	M	S	Q	K	S	N	L	C	D	F	G	V	S	G
PDB:3eqd:chainA:Q02750	L	G	A	G	N	G	G	V	A	K	I	V	M	E	M	S	Q	K	S	N	L	C	D	F	G	V	S	G
PDB:3w8q:chainA:Q02750	L	G	A	G	N	G	G	V	A	K	I	V	M	E	M	S	Q	K	S	N	L	C	D	F	G	V	S	G
sp:Q02750:MP2K1_HUMAN	L	G	A	G	N	G	G	V	A	K	I	V	M	E	M	S	Q	K	S	N	L	C	D	F	G	V	S	G
tr:A4QPA9:A4QPA9_HUMAN	L	G	A	G	N	G	G	V	A	K	I	V	M	E	M	S	Q	K	S	N	L	C	D	F	G	V	S	G
sp:Q01986:MP2K1_RAT	L	G	A	G	N	G	G	V	A	K	I	V	M	E	M	S	Q	K	S	N	L	C	D	F	G	V	S	G
tr:Q3TMJ8:Q3TMJ8_MOUSE	L	G	A	G	N	G	G	V	A	K	I	V	M	E	M	S	Q	K	S	N	L	C	D	F	G	V	S	G
sp:P31938:MP2K1_MOUSE	L	G	A	G	N	G	G	V	A	K	I	V	M	E	M	S	Q	K	S	N	L	C	D	F	G	V	S	G
sp:Q24324:DSOR1_DROME	L	G	S	G	N	G	G	V	A	K	I	V	M	E	M	S	L	K	S	N	L	C	D	F	G	V	S	G
sp:Q10664:MEK2_CAEEL	L	G	H	G	N	G	G	V	A	K	V	V	M	E	M	S	I	K	S	N	L	C	D	F	G	V	S	G
sp:P08018:PBS2_YEAST	L	G	H	G	N	Y	G	V	A	K	V	V	M	E	M	S	K	K	T	N	L	C	D	F	G	V	S	G

	whole protein		domain-based		contact-based	
protein	ident	simil	ident	simil	ident	simil
PDB:3eqc:chainA:Q02750	1.0	1.0	1.0	1.0	1.0	1.0
PDB:3eqd:chainA:Q02750	1.0	1.0	1.0	1.0	1.0	1.0
PDB:3w8q:chainA:Q02750	1.0	1.0	1.0	1.0	1.0	1.0
sp:Q02750:MP2K1_HUMAN	1.0	1.0	1.0	1.0	1.0	1.0
tr:A4QPA9:A4QPA9_HUMAN	1.0	1.0	1.0	1.0	1.0	1.0
sp:Q01986:MP2K1_RAT	0.99	1.0	1.0	1.0	1.0	1.0
tr:Q3TMJ8:Q3TMJ8_MOUSE	0.99	1.0	1.0	1.0	1.0	1.0
sp:P31938:MP2K1_MOUSE	0.99	1.0	1.0	1.0	1.0	1.0
sp:Q24324:DSOR1_DROME	0.6	0.82	0.75	0.93	0.93	0.93
sp:Q10664:MEK2_CAEEL	0.47	0.74	0.62	0.88	0.89	0.93
sp:P08018:PBS2_YEAST	0.2	0.36	0.45	0.74	0.82	0.88

Dsor1 (FBgn0010269) associated phenotypes
dominant, lethal - all die before end of pupal stage, neuroanatomy defective, polyphasic, some die during immature adult stage, some die during pupal stage

(Information from FlyBase)

Dsor1 (UniProt:Q24324) annotation
Function: Required downstream of Raf in the sevenless (sev), torso (tor), and Drosophila EGF receptor homolog (DER) signal transduction pathways. Involved in both positive regulation (at the posterior terminus) and negative regulation (at the anterior domain) of tll, as in other terminal class gene products, maybe via the ERK-A kinase. (PubMed:8381718).
Developmental stage: Expressed both maternally and zygotically. (PubMed:8381718).
Ptm: Phosphorylation on Ser/Thr by MAP kinase kinase kinases regulates positively the kinase activity.

(Information from UniProt)

mek-2 (WBGene00003186) associated phenotypes
AWA odorant chemotaxis defective, AWC odorant chemotaxis defective, bacterially unswollen, constipated, germ cell arrest, germ nuclei rachis, lethal, metabolic pathway variant, no oocytes, pachytene region organization variant, pathogen susceptibility increased, rod like larval lethal, sterile, vulval cell lineage variant, vulvaless

(Information from WormBase)

mek-2 (UniProt:Q10664) annotation
Function: Functions in the let-60 Ras signaling pathway; acts downstream of lin-45 raf kinase, but before the sur-1/mpK-1 gene product in controlling vulval cell differentiation.
Enzyme regulation: Activated by tyrosine and threoninephosphorylation catalyzed by MAP kinase kinase kinases.

(Information from UniProt)

Protein-tyrosine kinase 2-beta

Best gene implication in ageing for this target family came from gene Q14289 via mapping the annotation from Ensembl ENSG00000120899 via mapping the annotation from EntrezGene 2185 via mapping the annotation from GenAgeHuman 0165 annotated in GenAge release 17.

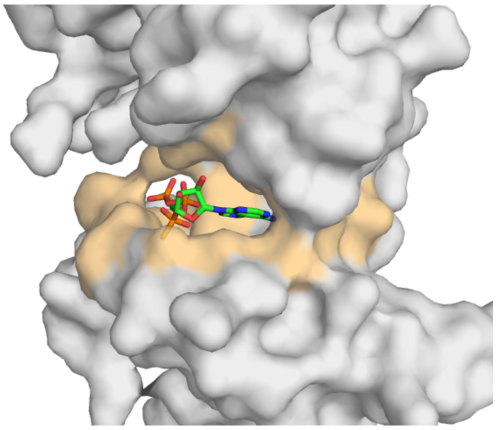

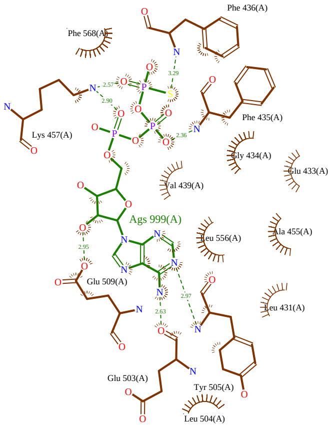

protein	amino acids contacts (binding site)
PDB:3fzp:chainA:Q14289	L E G F F V A K E E L Y E L D F
tr:E9PBI4:E9PBI4_HUMAN	L E G F F V A K E E L Y E L D F
sp:Q14289:FAK2_HUMAN	L E G F F V A K E E L Y E L D F
sp:P70600:FAK2_RAT	L E G F F V A K E E L Y E L D F
tr:E9Q2A6:E9Q2A6_MOUSE	L E G F F V A K E E L Y E L D F
sp:Q9QVP9:FAK2_MOUSE	L E G F F V A K E E L Y E L D F
tr:Q3UDE9:Q3UDE9_MOUSE	L E G F F V A K E E L Y E L D F
tr:K7QD41:K7QD41_MOUSE	L E G F F V A K E E L Y E L D F
tr:Q2TGI0:Q2TGI0_DROME	I V G Q F V A K E E L A E L D F
tr:Q9U5Y2:Q9U5Y2_DROME	I V G Q F V A K E E L A E L D F
tr:E1JGM8:E1JGM8_DROME	I V G Q F V A K E E L A E L D F
tr:B8A418:B8A418_DROME	I V G Q F V A K E E L A E L D F
tr:Q5BIG9:Q5BIG9_DROME	I V G Q F V A K E E L A E L D F
tr:Q0E917:Q0E917_DROME	I V G Q F V A K E E L A E L D F
tr:Q9U531:Q9U531_DROME	I V G Q F V A K E E L A E L D F
tr:Q9U472:Q9U472_DROME	I V G Q F V A K E E L A E L D F
tr:B7YZL9:B7YZL9_DROME	I V G Q F V A K E E L A E L D F
tr:Q8T879:Q8T879_CAEEL	I G G Q F V A K E E L A E L D F
tr:Q95YD4:Q95YD4_CAEEL	I G G Q F V A K E E L A E L D F

	whole protein		domain-based		contact-based	
protein	ident	simil	ident	simil	ident	simil
PDB:3fzp:chainA:Q14289	1.0	1.0	1.0	1.0	1.0	1.0
tr:E9PBI4:E9PBI4_HUMAN	0.54	0.56	1.0	1.0	1.0	1.0
sp:Q14289:FAK2_HUMAN	1.0	1.0	1.0	1.0	1.0	1.0
sp:P70600:FAK2_RAT	0.96	0.99	0.98	0.99	1.0	1.0
tr:E9Q2A6:E9Q2A6_MOUSE	0.91	0.95	0.98	0.99	1.0	1.0
sp:Q9QVP9:FAK2_MOUSE	0.95	0.99	0.98	0.99	1.0	1.0
tr:Q3UDE9:Q3UDE9_MOUSE	0.95	0.98	0.98	0.99	1.0	1.0
tr:K7QD41:K7QD41_MOUSE	0.95	0.99	0.98	0.99	1.0	1.0
tr:Q2TGI0:Q2TGI0_DROME	0.14	0.22	0.51	0.76	0.75	0.79
tr:Q9U5Y2:Q9U5Y2_DROME	0.25	0.59	0.51	0.76	0.75	0.79
tr:E1JGM8:E1JGM8_DROME	0.25	0.59	0.51	0.76	0.75	0.79
tr:B8A418:B8A418_DROME	0.25	0.59	0.51	0.76	0.75	0.79
tr:Q5BIG9:Q5BIG9_DROME	0.25	0.59	0.5	0.76	0.75	0.79
tr:Q0E917:Q0E917_DROME	0.25	0.59	0.51	0.76	0.75	0.79
tr:Q9U531:Q9U531_DROME	0.25	0.59	0.51	0.76	0.75	0.79
tr:Q9U472:Q9U472_DROME	0.25	0.6	0.51	0.76	0.75	0.79
tr:B7YZL9:B7YZL9_DROME	0.2	0.48	0.51	0.76	0.75	0.79
tr:Q8T879:Q8T879_CAEEL	0.2	0.56	0.46	0.82	0.75	0.81
tr:Q95YD4:Q95YD4_CAEEL	0.21	0.6	0.46	0.82	0.75	0.81

Fak (FBgn0020440) associated phenotypes

neuroanatomy defective, neurophysiology defective

(Information from FlyBase)
